# Stage-Specific Profiling of Transforming Growth Factor-β, Fibroblast Growth Factor and Wingless-int Signaling Pathways during Early Embryo Development in The Goat 

**DOI:** 10.22074/cellj.2016.3837

**Published:** 2016-01-17

**Authors:** Pouria HosseinNia, Mojtaba Tahmoorespur, Sayyed Morteza Hosseini, Mehdi Hajian, Somayeh Ostadhosseini, Mohammad Reza Nasiri, Mohammad Hossein Nasr-Esfahani

**Affiliations:** 1 Department of Animal Science, Faculty of Agriculture, Ferdowsi University of Mashhad, Mashhad, Iran; 2 Department of Reproductive Biotechnology, Reproductive Biomedicine Research Center, Royan Institute for Biotechnology, ACECR, Isfahan, Iran

**Keywords:** Goat, Gene Expression, TGF-β, FGF, WNT

## Abstract

**Objective:**

This research intends to unravel the temporal expression profiles of genes in- volved in three developmentally important signaling pathways [transforming growth factor-β (TGF-β), fibroblast growth factor (FGF) and wingless/int (WNT)] during preand peri-implan- tation goat embryo development.

**Materials and Methods:**

In this experimental study, we examined the transcripts that encoded the ligand, receptor, intracellular signal transducer and modifier, and the down- stream effector, for each signaling pathway. *In vitro* mature MII oocytes and embryos at three distinctive stages [8-16 cell stage, day-7 (D7) blastocysts and day-14 (D14) blas- tocysts] were separately prepared in triplicate for comparative real-time reverse tran- scriptase polymerase chain reaction (RT-PCR) using the selected gene sets.

**Results:**

Most components of the three signaling pathways were present at more or less stable levels throughout the assessed oocyte and embryo developmental stages. The transcripts for TGF-β, FGF and WNT signaling pathways were all induced in unfertilized MII-oocytes. However, developing embryos showed gradual patterns of decrease in the activities of TGF-β, FGF and WNT components with renewal thereafter.

**Conclusion:**

The results suggested that TGF-β, FGF and WNT are maternally active signaling pathways required during earlier, rather than later, stages of preand peri- implantation goat embryo development.

## Introduction

Pre-implantation embryo development is a characteristic feature of mammalian embryo development which encompasses a series of crucial events suchas the transition from oocyte to embryo, first cell divisions, and establishment of cellular contacts. These processes are under strict control of spatial and temporal regulation of gene expression, cell polarization, and cell-cell interactions (1). 

The transcriptional circuitry that regulates embryo development comprises several hundred genes responsible for cell division, growth, differentiation, polarity, and apoptosis of embryonic cells. By combining several functions, such as cross-linking and other interactions, these genes provide pathways to form a complicated network of interactions that take shape in the context of various cell-signaling pathways which include fibroblast growth factors (FGF), mitogen-activated protein kinase (MAPK), phosphatidylinositol 3-kinase (PtdIns3K)⁄protein kinase B (PKB), also known as Akt and Janus-activated kinase (JAK)⁄signal transducer and activator of transcription (STAT), the wingless/int (WNT)⁄b-catenin pathway, notch, bone morphogenetic protein (BMP)-Smad, and hedgehog (1).

Pluripotency and self-renewal in the absence of differentiation are three fundamental traits of embryonic stem cells (ESCs) which are mostly maintained by the core transcription triad-*OCT4, SOX2,* and *NANOG* (2). Importantly, it has been established that the pluripotency transcription triad is highly responsive to upstream and downstream signals induced by WNT, transforming growth factor-β (TGF-β) and FGF signaling pathways. A functional WNT signaling system operates in the pre-implantation embryo and activation of the canonical pathway affects embryonic development in bovines (3), ESC self-renewal in humans and mice (4), as well as tumor progression (5). It is well established that phosphorylation inhibition of TGF-β signaling by SB(4-(5-Benzol[1,3] dioxol-5-yl-4-pyrldin-2-yl-1H-imidazol-2-yl)- benzamide hydrate, 4-[4-(1,3-Benzodioxol-5-yl)- 5-(2-pyridyridyl)-1H-imidazol-2-yl]-benzamide hydrate) supports mouse ESC self-renewal in the differentiation (6). The growth of primed stem cells are dependent on the FGF signaling pathway and notably, dual inhibition of extracellular signalregulated protein kinases 1 and 2 (ERK1/2) and glycogen synthase kinase 3 (GSK3), designated as 2i, has been shown to improve the efficacy of ESC-derivation in mice (7).

Despite two decades of effort, derivation of authentic ungulate ESCs remains challenging for embryologists. To date, ESCs have been successfully isolated only in rodents and primates. A clear understanding of the signaling pathways that regulate early embryo development will greatly benefit the current understanding of developmental biology and approaches to capture pluripotent stem cells *in vitro*. The current study has attempted to investigate the dynamics of expression of components from the three developmental signaling pathways (WNT, TGF-β, and FGF) at four distinctive stages of goat embryo development: i. Unfertilized *in vitro* mature (MII)-oocytes, ii. 8-16 cell stage which coincides with the stage concomitant with zygote genome activation in the goat, iii. Day-7 (D7) blastocysts and IV. Day-14 (D14) blastocysts.

## Materials and methods

### Chemicals and media

Unless otherwise stated, all chemicals were obtained from Sigma Chemical Co. (USA) and media from Gibco (Grand Island, USA).

#### Selection of gene sets

Due to the lack of sufficient data in the goat species, we searched related studies in humans (8), mice (9), bovine (10) and porcine (11) for the conserved upstream and downstream components of the TGF-β, FGF, and WNT signaling pathways. Accordingly, we selected 4 transcripts for the TGF-β (*Bmpr1a, Alk4, Sdma1* and 5, Id3), 4 transcripts for the FGF (*Lifr1, Akt, Fgf4, Erk1, Cdc25a*) and 3 transcripts for the WNT (*Fzd, Ctnnb, c-Myc*) signaling pathways. The genes involved in both the core pluripotency triad (*Oct4, Nanog, Sox2*) and cell lineage commitment (*Rex1, Cdx2, Gata4*) were also considered. We took into consideration the lack of a previous report or database on gene sequences of many of these genes and designed the primers according to the conserved regions of these markers in bovine, ovine, humans and mice. For *Erk1, Alk4, Bmpr1, Fgfr4* and *Lifr1*, portions of the cDNA were initially sequenced and registered in the NCBI site under the following accession numbers:

KC687077 (http://www.ncbi.nlm.nih.gov/nuccore/KC687077),
KF039752 (http://www.ncbi.nlm.nih.gov/nuccore/KF039752),
KF039753 (http://www.ncbi.nlm.nih.gov/nuccore/KF039753),
KF039754 (http://www.ncbi.nlm.nih.gov/nuccore/KF039754), and
KF356183 (http://www.ncbi.nlm.nih.gov/nuccore/KF356183).

Specific primers were subsequently designed from these recognized sequences (Table 1).

**Table 1:** Specific real-time primers designed for gene sequences


Gene	Primer sequences	Length of PCR product	TM

*Lifr1*	F: ATTTTTCGGTGTATGGGTGC	117	56
	R: CAGATGTATCCTCAACGGTA		
*Bmpr1*	F: CCTGTTCGTCGTGTCTCAT	116	58
	R: CTGGTGCTAAGGTTACTCC		
*Alk4*	F: TCTCCAAGGACAAGACGCTC	152	62
	R: ACGCCACACTTCTCCAAACC		
*Smad1*	F: TCACCATTCCTCGCTCCCT	140	60
	R: AAACTCGCAGCATTCCAACG		
*Smad5*	F: ACAGCACAGCCTTCTGGTTC	136	60
	R: GGGGTAGGGACTATTTGGAG		
*Id3*	F: CGGCTGAGGGAACTGGTA	198	58
	R: CCTTTGGTCGTTGGAGATG		
*Ctnnb*	F: AGTGGGTGGCATAGAGG	160	54
	R: CACAGGTAGCCCGTAG		
*Akt*	F: TTCAGCAGCATCGTGTGGCA	98	60
	R: TCATCAAAATACCTGGTGTCCG		
*Oct4*	F: GCCAGAAGGGCAAACGAT	96	56
	R: GAGGAAAGGATACGGGTC		
*Rex1*	F: GCAGCGAGCCCTACACAC	94	61
	R: ACAACAGCGTCATCGTCCG		
*Fzd*	F: CATCGGCACTTCCTTTATCC	89	59
	R: GCTTGTCCGTGTTCTCCC		
*C-myc*	F: CAACACCCGAGCGACACC	160	61
	R: GCCCGTATTTCCACTATCCG		
*Sox2 *	F: ATGGGCTCGGTGGTGA	182	54
	R: CTCTGGTAGTGCTGGGA		
*Fgfr4*	F: GCTGACTGGTAGGAAAGG	193	56
	R:AGTGGCTGAAGCACATCG		
*Nanog*	F: GATTCTTCCACAAGCCCT	137	54
	R:TCATTGAGCACACACAGC		
*Erk1*	F:TCAAGCCGTCCAACATCCT	204	58
	R:CGACCGCCATCTCAACC		
*Gata4*	F:TCCCCTTCGGGCTCAGTGC	128	64
	R:GTTGCCAGGTAGCGAGTTTGC		
*Cdx2*	F:CCCCAAGTGAAAACCAG	144	53
	R:TGAGAGCCCCAGTGTG		
*Cdc25a*	F:TGGCAAGCGTGTTATCGTG	119	58
	R:GGTAGTGGAGTTTGGGGTA		
*ACTB*	F:CCATCGGCAATGAGCGGT	146	60
	R:CGTGTTGGCGTAGAGGTC		


PCR; Polymerase chain reaction and TM; Melting temperature.

#### In vitro production of goat embryos

This experimental study was conducted from 2011-2014.We used 850 goat ovaries that hadbeen derived from local breed does (Isfahani, Najdi) immediately after slaughter. The procedure for *in vitro* production of goat embryos has been previously described (12). In brief, goat ovaries were used for *in vitro* maturation of cumulus-oocyte complexes (COCs) in tissue culture medium-199 (TCM199) plus 10% fetal calf serum) FCS, (2.5 mM sodium pyruvate, 100 IU/mL penicillin, 100 μg/mL streptomycin, 10 μg/mL follicle stimulating hormone (FSH), 10 μg/mL luteinizing hormone (LH), 1 μg/mL estradiol-17β, and 0.1 mM cysteamine under mineral oil for 20-22 hours at 39˚C, 5% CO_2_, and maximum humidity. Next, they were divided into six groups and placed in 20 μl droplets that consisted of a modified formulation of synthetic oviductal fluid (mSOF) (12) and maintained at 39˚C, 6% CO_2_, 5% O_2_, and maximum humidity for embryo development. The MII oocytes at 20-22 hours post-maturation, day 3 (D3) developing embryos at the 8-16 cell stage, and day 7 (D7) blastocysts were collected, washed three times in phosphate-buffered saline (PBS), collected in pools of 60 (oocytes), 35-40 (D3 developing embryos), and 20 (D7 blastocysts) in 500 μL microtubes that contained lysis buffer RLT. They were subsequently frozen and stored at -70˚C until RNA extraction. All oocyte and embryo pools used for RNA extractions were collected and analyzed in triplicate. This system of embryo development had adequate rates of in vitro embryo development with cleavage rates between 85 to 92% and blastocyst rates between 40-45%.

### Derivation of day-14 embryos

In order to extend the *in vitro* culture of goat blastocysts, we prepared a feeder later of caprine fetal fibroblasts (CFF) as described by Behboodi et al. (13). For this purpose, the CFF line was derived from three 40-day male fetuses surgically from donors. A single-cell suspension was prepared by mincing fetal tissue and culturing the tissue in Dulbecco’s modified eagle’s medium (DMEM) supplemented with 10% FBS, 0.25% amphotericin-B, 1% penicillin-streptomycin, and 1% gentamicin in 25 cm^2^ culture flasks at 37˚C and 6% CO_2_ until the appearance of a confluent monolayer from D4 onwards. The monolayer was trypsinized and further cultured for proliferation of the CFF source. Each passage took approximately 3-4 days until confluency. Passages 2-4 CFFs were treated with mitomycin (10 mg/mL) for 2 hours. Mitomycin treated cells were washed twice with DMEM and treated with trypsin-Ethylenediaminetetraacetic acid (EDTA) 0.25% (Supplemented by EDTA) and gently pipetted the confluent monolayer in order to obtain single cells. Cells were then seeded at 1×10^5^cells/ml in 100 μl DMEM drops in the vicinity of a feeder-free 100 μl droplet of DMEM supplemented with 10% FBS, 1% Lglutamine, 1% non-essential amino acids, and 0.1% β-mercaptoethanol under mineral oil. We transferred 5-6 D7 blastocysts to each 100 μl droplet of feeder-free DMEM. By the aid of the tip of a draw pipette glass, the DMEM drops that contained blastocysts were gently connected to their adjacent DMEM that had a CFF monolayer. This joined culture system provided the beneficial effects of a feeder layer for extended *in vitro* embryo culture, but prevented attachment and flattening of the elongating blastocysts. The joined droplets were refreshed every other day until D14 of embryo development when pools of 7-10 well-developed spherical D14 embryos were pooled for RNA extraction as previously described.

### RNA extraction and real time-polymerase chain reaction

The procedure for quantitative real-time PCR (qRT-PCR) has been previously described (14). In brief, total RNA from MII-oocytes, 8-16 (D3), blastocysts (D7) and elongating embryos (D14) was extracted with the RNeasy Micro kit (Qiagen, Canada) followed by treatment with DNase I (Ambion, Canada) according to the manufacturer’s protocol. RNA quality and quantity were determined using a WPA Biowave spectrophotometer (Cambridge, United Kingdom). For reverse transcription, 10 μl of total RNA was used in a final volume of a 20 μl reaction that contained 1 μl of random hexamer, 4 μl RT buffer (10x), 2 μl of dNTP, 1μl of RNase inhibitor (20 IU), and 1μl of reverse transcriptase (Fermentas, Canada). Reverse transcription was carried out at 25˚C for 10 minutes, 42˚C for 1 hour and 70˚C for 10 minutes.

### Quantitative analysis of transcripts by real time-polymerase chain reaction

The transcripts abundance of the mentioned genes (Table 2) and *ACTB* as the housekeeping gene were analyzed with real-time RTPCR. Briefly, total RNA from the oocytes, D3 embryos, D7 blastocysts, and D14 blastocysts were extracted. Each of the RNA samples was used for cDNA synthesis. Real-time RT-PCR was carried out using 1 μl of cDNA (50 ng), 5 μl of the SYBR Green/0.2 μl ROX qPCR Master Mix (2X, Fermentas, Germany) and 1 μl of forward and reverse primers (5 pM) adjusted to a total volume of 10 μl using nuclease-free water. The primer sequences, annealing temperatures and the size of amplified products are shown in table 1.

### Statistical analysis

Statistical significance analysis was considered to be P<0.05 and determined by the two-tailed Fisher’s exact test in SPSS software version 20 for developmental data using two-tailed student’s t test with equal variance for cell counts and realtime PCR data.

**Table 2:** Detailed results of relative expressions of goat embryo during developmental stages by quantitative real-time PCR (qRT-PCR)


Gene	MII oocytes	8-16 cell	D7 blastocysts	D14 blastocysts

*Lifr1*	1^a^	0.402^a^	0.014^b^	0.001^b^
*Bmpr1*	1^a^	0.227^b^	0.015^b^	0.005^b^
*Alk4*	1^a^	0.242^b^	0.002^c^	0.002^c^
*Smad1*	1^a^	0.332^b^	0.028^c^	0.014^c^
*Smad5*	1^a^	0.592^b^	0.002^c^	0.002^c^
*Id3*	1^a^	0.118^b^	0.0001^c^	0.0001^c^
*Fzd*	1^b^	14.16^a^	0.170^c^	0.240^c^
*Ctnnb*	1^a^	0.327^b^	0.012^c^	0.004^c^
*c-Myc*	1^b^	60.00^a^	0.420^bc^	0.080^c^
*Fgfr4*	1^c^	218.0^a^	3.000^c^	9.000^b^
*Erk1*	1^a^	0.189^b^	0.004^b^	0.126^b^
*Cdc25a*	1^b^	2.884^a^	0.003^c^	0.016^c^
*Akt*	1^a^	0.640^a^	0.040^b^	0.050^b^
*Oct4*	1^a^	1.000^a^	0.280^b^	0.010^c^
*Rex1*	1^a^	0.718^b^	0.004^c^	0.010^c^
*Sox2*	1^b^	22.15^a^	0.080^c^	0.060^c^
*Nanog*	1^c^	6.700^b^	0.600^c^	12.30^a^
*Gata4*	1^b^	0.280^c^	0.020^c^	1.780^a^
*Cdx2*	1^b^	0.530^bc^	0.190^c^	4.220^a^


Significant difference at P<0.05%. ^a, b, c^; No significant differences between the same letter and D; Day.

## Results

### In vitro embryo development

The system used in this study for *in vitro* goat embryo development supported over 90% *in vitro* maturation based on the assessment of first polar body extrusion with cleavage rates from 85-92% and blastocyst rates between 40-45%. The quality of embryos were quite reasonable. In our routine system of goat embryo development, approximately 50% of *in vitro* developed blastocysts resulted in successful pregnancy and delivery of healthy kids (15). The culture system used to culture the blastocysts provided the beneficial effects of a feeder layer for extended in vitro embryo culture and prevented both attachment and flattening of the elongating blastocysts. From 50- 65% of the developed blastocysts progressed to the elongation stage.

### Gene expression results

Table 2 lists the detailed results of real-time RTPCR of the 19 genes for each of the *in vitro* goat embryo developmental stages. In order to better illustrate the dynamics of each gene in a certain signaling pathway, we have adjusted the expression level of each gene to 100%; the expression levels at other time points were normalized to the peak level percentage (16). The transformed data were subsequently used to extrapolate the expression status of different components of the TGF-β, FGF, and WNT signaling pathways.

### Transforming growth factor-β signaling pathway

**Fig.1:**
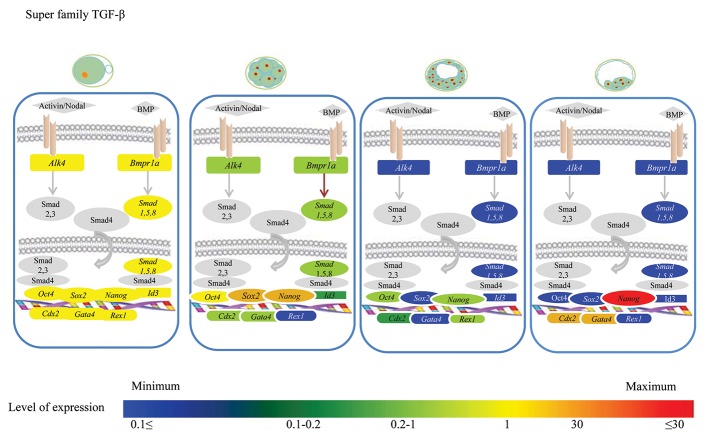
Comparison of transforming growth factor-β (TGF-β) mRNA transcript expression during pre-implantation developmental stages. Oocyte stage gene expression as calibrator. All core pluripotency markers, with the exception of *Nanog* reduced from the 8-16 cell stage to the day-14 (D14) blastocyst stage. These factors possibly promote or antagonize interconversion between differentiation and pluripotency.

Figure 1 shows the stage-specific expression status of different components of the TGF-β signaling pathway in association with the core pluripotency triad. As shown, MII-oocytes contained the highest transcript amounts of *Bmpr1, Alk4, Smad1, Smad5,* and *Id3* compared to the other embryo stages. At the 8-16 cell stage, the relative abundance of all transcription factors decreased by 10% (*Id3*) to 60% (*Smad5*) of the initial abundances observed in the MII-oocytes. Further development of the embryos to D7 blastocysts was concomitant with the minimum relative abundances of these transcripts compared to the MII and 8-16 cell stages. The expression status of D14 embryos for different components of the TGF-β signaling was the same as for D7 blastocysts.

### Fibroblast growth factor signaling pathway

**Fig.2:**
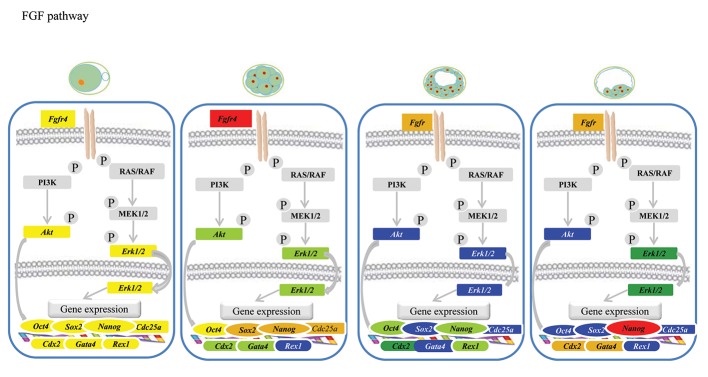
Expression of genes involved in the fibroblast growth factor (FGF) signaling pathway during goat pre-implantation development. qRT-PCR indicates low or lack of *Rex1* expression during the8-16 cell stage; *Akt, Gata4, Sox2* and *Erk1/2* in the day-7 (D7) blastocyst stage; and *Akt, Rex1, Oct4* and *Sox2* in the day-14 (D14) blastocyststage.

Figure 2 shows the stage-specific expression status of different components of the FGF signaling pathway in association with the core pluripotency triad. As shown, there were two different expression patterns observed for components of the FGF signaling pathway. In the first pattern, MII-oocytes had the highest transcript abundances of *Erk1, Bmpr1* and *Akt* compared to the other embryo stages. At the 8-16 cell stage, the relative abundances of all transcription factors decreased from 20% (*Erk1*) to 60% (*Akt*) of the initial abundances observed in the MII-oocytes. Further development of the embryos to D7 blastocysts was concomitant with the minimum relative abundances of these transcripts compared to the MII and 8-16 cell stages. Development to D14 blastocysts did not change their expressions compared to D7 blastocysts. In the second expression pattern, *Fgfr4* and *Cdc25a* had maximum expression levels at the 8-16 cell stage compared to the other stages. Embryos that developed to D14 showed a medium increase in transcription of *Fgfr4*.

### Wingless/int signaling pathway

**Fig.3:**
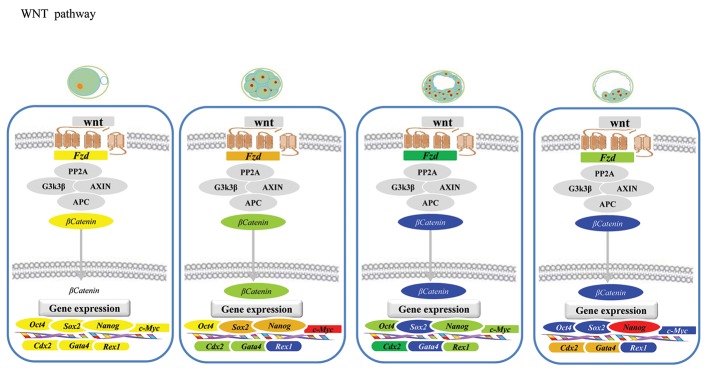
Wingless/int (WNT) signaling in goat pre-implantation embryos. Expressions of *c-Myc*, *Nanog* and *Sox2* were detected at higher levels in 8-16 cell embryos. *Nanog*, *Cdx2* and *Gata4* expressed at higher levels in the day-14 (D14) developmental stage. Values are of fold change calculated by using 2^-ΔΔCT^.

Figure 3 shows the stage-specific expression status of different components of the WNT signaling pathway in association with the core pluripotency triad. As shown, *Fzd* and *c-Myc* both had a similar pattern in which a peak of expression was observed at the 8-16 cell stage, whereas their expressions either before (MII-oocyte) or after (D7 and D14 blastocysts) were at minimum levels. *Ctnnb* had the highest transcript level at the MII-oocyte stage which substantially decreased atthe 8-16 cell stage. Further development to D7 and D14 blastocysts did not induce a change in transcription when compared to the 8-16 cell stage.

### Core pluripotency triad

*Oct4* showed its highest transcript abundance in MII-oocytes which persisted until the 8-16 cell stage but sharply decreased at the D7 and D14 developed blastocysts stages. The highest abundance of Sox2 was observed at the 8-16 cell stage compared to its lowest transcript abundance in the MIIoocyte stage. And also Sox2 had the lowest amount of transcript in D7 and D14 blastocysts stage compared to other stages. The lowest transcript levels of *Nanog* were found at MII-oocytes and D7 blastocysts with a medium burst in transcription at the 8-16 cell stage. Development to the D14 blastocyst was concomitant with a sharp increase in transcription activation of *Nanog* to its highest level at the D14 blastocyst stage.

### Lineage specific markers

Figures 1-3 represent the stage-specific expression status of three specific linage markers. As shown, both *Gata4* and *Cdx2* showed their lowest expression levels in D7 blastocysts, whereas the highest expression levels were at the D14 blastocyst stage. Their transcript abundances at MII-oocyte stage were moderate but decreased to 10% of the levels observed in D14. In contrast, the highest transcript level of *Rex1* was observed in MIIoocytes with a 30% decrease in 8-16 cell stage embryos and significant decrease to its minimum levels in D7 and D14 blastocysts.

## Discussion

Mammalian development is based upon the capacity of pluripotent inner cell mass (ICM) cells for specification to over 200 cell lineages. This capacity has been captured *ex vivo* in rodents, then in primates through extrapolation of molecular pathways responsible for pluripotency in ICM cells (17). However, despite two decades of effort, derivation of authentic ungulate ESC remains an ongoing challenge for embryologists. Outstanding issues that include species-specific differences in pre-implantation embryo development, pluripotency pathways, and culture conditions may hinder the efforts to establish authentic ungulate ESC lines (17, 18). Here, we have attempted to unravel the transcriptional status of genes involved in three principal signaling pathways in association with pluripotency and cell linage commitment genes.

Generally, a signaling pathway is considered to be active if multiple components of signal transduction are expressed and if their patterns of expression of these genes are developmentally regulated (16). For the TGF-β, FGF, and WNT pathways, we have observed that the transcripts which encode ligands, receptors (*Fgfr4, Lifr1, Bmpr1a, Alk4,* and *Fzd*), intracellular signal transducers and modifiers (*Ctnnb, Erk1, Akt, and Smad1, 5*), and nuclear effectors (*c-Myc, Id3, Cdc25a, Oct4, Sox2, Nanog, Cdx2, Gata4,* and *Rex1*) were present. However, a number of signal transduction transcripts were more or less abundant with great fluctuations throughout preand peri-implantation development. Accordingly, while *Oct4, Erk1*, and *Nanog* continuously expressed throughout the analysis, *Lifr1, Bmpr1, Alk4, Id3, Ctnnb, Smad2, 3, Akt,* and *Rex1* were abundant during the earliest stages of development and substantially decreased thereafter. In contrast, *Gata4* and *Cdx2* transcripts were highly abundant during the later stages of embryo development. *Fzd, cMyc, Cdc25a, Sox2,* and *Fgfr4* highly expressed during the 8-16 cell stage but were at minimum levels before and after this stage. These patterns of gene expression corresponded to the zygote genome activation in 8-16 cell stages ofgoat species.

The TGF-β superfamily is considered a major regulator of cell growth, pluripotency/differentiation, and tumor suppression in the context of a large variety of biological systems. The main body of the TGF-β superfamily is composed of nearly 30 growth and differentiation factors that include TGF-β ligands (TGF-βs, Activins, Nodals) and BMP ligands (BMP-2, 4-7, MIS) (19). The interplay within TGF-β and between TGF-β, BMP and other signaling pathways is a stringent mechanism for the definition of the stem cell fate (20). Smad and co-Smad transcription factors (Smads) along with their inhibitors (I-Smads) are the main mediators. In this context, we have found that different components of TGF-β were highly abundant in MII-oocytes and subsequently decreased with no trend of re-initiation during transcription. This might suggest that TGF-β signal transduction was a maternally regulated system which might be in a relatively repressive state during early stages of embryo development in the goat.

As the key players of proliferation and differentiation in a wide range of cells and tissues, the FGF family of growth factors are amongst the most studied pathway for *ex vivo* induction and maintenanceof pluripotent stem cells (PSCs). It has been well established that ESCs that are null for FGF4 or those cultured in the presence of FGF receptor inhibitors such as PD0325901 are refractory to BMP-induced differentiation. Importantly, even in the absence of FGF4 in Fgf4-null cells, provision of FGF protein can restore the capacity of ESCs for differentiation. We have found that components of FGF signal transduction were differently expressed during preand peri-implantation in the goat. Highest expressions of Fgfr4 and Cdc25 were observed at the 8-16 cell stage. *Erk1, Lifr1* and *Akt* were highly abundant in MII-oocytes and decreased thereafter. Possibly, FGF signal transduction might not be active during early stages of embryo development, in particular D7 blastocysts, in the goat. 

Double inhibition of FGF (by small molecule PD0325901) and GSK3 (by small molecule CHIR) can promote ground state pluripotency, which in turn reflect the importance of an FGF inductive signaling (21). Harris et al. (22) applied 2i throughout bovine *in vitro* development and observed accelerated blastocyst development, increase numbers of ICM and TE cells, and notably increased expressions of *Nanog* and *SOX2*, with repressed putative hypoblast marker *Gata4*. These findings mightsupport the suggestion that suppression of the FGF pathway could pose an active pluripotency state in goat D7 and D14 blastocysts without the need for 2i inhibition. Further studies to clarify this issue would require precise detection of the expression state of the FGF signaling pathway at the protein level. 

The WNT pathways comprise a wide variety of conserved glycoproteins that act as regulators of cancer, embryonic development, and cell fate/ proliferation. WNT pathway integrates signals at several points of the cascade with other pathways, including FGF and TGF-β (4,23). Activation of the WNT canonical pathway is important for selfrenewal in human and mice ESCs (4), in addition to tumor progression (5). Here, we have observed peak *Fzd* receptor transcripts at the 8-16 cell stage which quenched during D7 and D14 blastocyst development. Although *Ctnnb* transcripts were highly abundant in MII-oocytes, they sharply decreased during later developmental stages. The absence of *Ctnnb* hindered its translocation into the nucleus. The 2-amino-4-( 3,4-(methylenedioxy) benzylamino)-6-(3-methoxyphenyl)pyrimidine (AMBMP) and Dickkopf-related protein 1 (DKK1) are known activators and inhibitors of theWNT pathway. Interestingly, AMBMP-mediated activation of WNT signal transduction has decreased *in vitro* development of bovine embryos and reduced numbers of ICM and TE. It has been shown that day 6 bovine morula express 16 WNT genes and other genes involved in WNT signaling. They concluded that activation of the canonical WNT pathway inhibited bovine embryonic development (3). Therefore, our observation that a downstream signal of WNT was repressed in the goat blastocyst might suggest a poised state of developmental capacity in goat embryos compared to bovine embryos. 

## Conclusion

This study has provided the first set of data on the transcriptional states of TGF-β, FGF and WNT which are well established regulators of pluripotency and differentiation during preand peri-implantation goat embryo development. The resultant data suggested that TGF-β, FGF and WNT were highly active in unfertilized MII-oocytes.Their activities were repressed during subsequent stages of embryo development. This information suggested that TGF-β, FGF and WNT were maternally active signaling pathways required during earlier rather than later stages of preand peri–implantation embryo development in the goat. These data would increase our knowledge of signaling pathways that control early embryo development in this valuable farm species and would greatly benefit current chemical approaches used for manipulation of the ICM fate *in vitro*. 
